# Pathology of Camel Tuberculosis and Molecular Characterization of Its Causative Agents in Pastoral Regions of Ethiopia

**DOI:** 10.1371/journal.pone.0015862

**Published:** 2011-01-24

**Authors:** Gezahegne Mamo, Gizachew Bayleyegn, Tesfaye Sisay Tessema, Mengistu Legesse, Girmay Medhin, Gunnar Bjune, Fekadu Abebe, Gobena Ameni

**Affiliations:** 1 Aklilu Lemma Institute of Pathobiology, Addis Ababa University, Addis Ababa, Ethiopia; 2 Faculty of Veterinary Medicine, Addis Ababa University, Debre Zeit, Ethiopia; 3 Department of General Practice and Community Medicine, Faculty of Medicine, Institute of Health and Society, University of Oslo, Oslo, Norway; University of Hyderabad, India

## Abstract

A cross sectional study was conducted on 906 apparently healthy camels slaughtered at Akaki and Metehara abattoirs to investigate the pathology of camel tuberculosis (TB) and characterize its causative agents using postmortem examination, mycobacteriological culturing, and multiplex polymerase chain reaction (PCR), region of difference-4 (RD4)-based PCR and spoligotyping. The prevalence of camel TB was 10.04% (91/906) on the basis of pathology and it was significantly higher in females (χ^2^ = 4.789; P = 0.029). The tropism of TB lesions was significantly different among the lymph nodes (χ^2^ = 22.697; P = 0.002) and lung lobes (χ^2^ = 17.901; P = 0.006). Mycobacterial growth was observed in 34% (31/91) of camels with grossly suspicious TB lesions. Upon further molecular characterization using multiplex PCR, 68% (21/31) of the colonies showed a positive signal for the genus *Mycobacterium*, of which two were confirmed *Mycobacterium bovis (M. bovis)* by RD4 deletion typing. Further characterization of the two *M. bovis* at strains level revealed that one of the strains was SB0133 while the other strain was new and had not been reported to the *M. bovis* database prior to this study. Hence, it has now been reported to the database, and designated as SB1953. In conclusion, the results of the present study have shown that the majority of camel TB lesions are caused by mycobacteria other than *Mycobacterium tuberculosis* complex. And hence further identification and characterization of these species would be useful towards the efforts made to control TB in camels.

## Introduction

Pastoral production system accounts for the livelihood of 50–100 million people in developing countries and 60% of this population lives in more than 21 African countries confined to the most arid regions of the continent [1], [2]. In eastern Africa, Ethiopia has the largest pastoralist population (7–8 millions) representing around 20 ethnic groups [3]. The major ethnic groups in Ethiopia are Somalis, Afar, Kereyu and Borena pastoral communities occupying the Eastern and southern lowlands of the country. Pastoralist depends on livestock for their livelihood, moving seasonally from place to place in search of water and pasture for their animals [4]. The dromedary camel (*Camelus dromedarius*), which is a versatile animal capable of living in harshly semi-arid and arid areas of the world, is extremely important for livelihood of pastoral communities through provision of milk, meat and draft power for transportation of goods. In pastoral communities of Afar, Somali and Borena, camels are kept almost entirely for milk production [5]. In these communities, camel milk is consumed raw, and this habit combined with close physical contact with their animals create a potential public health concern for transmission of zoonotic diseases such as tuberculosis (TB) from animals to the pastoralist.

Although, the extent of TB has been well documented in humans and most domestic animals, very little is known about the pathology and cause of camel TB in pastoral areas of the world. Camel TB has been reported in Egypt [6], United Arab Emirates [7], [8], Pakistan [9], and Australia [10]. *Mycobacterium tuberculosis (M. tuberculosis), Mycobacterium bovis* (*M. bovis*), and atypical mycobacteria such as *Mycobacterium kansasii* (*M. kansasii*), *Mycobacterium aquae* (*M. aquae*), *Mycobacterium fortuitum* (*M. fortuitum*) and *Mycobacterium smegmatis* (*M. smegmatis*) have been isolated in camel as causative agents of camel TB [8], [11]. In Ethiopia, except one report indicating the existence of camel TB [12], there is a large paucity of information on the pathology and the causative agent of TB in camels of pastoral regions of the country. Therefore, investigation of the pathology of camel TB and identification of its causative agents is important to encourage the effort in the control of the disease and reduce its risk of zoonosis to the pastoralist community of Ethiopia. The present study, therefore, was designed to investigate the pathology of camel TB and identify the causative agent using molecular tools.

## Materials and Methods

### Study Animals

The cross sectional study was carried out on 906 apparently healthy male (n = 535) and female (n = 371) slaughtered camels. The camels slaughtered were brought to Akaki (Addis Ababa) and Metehara Abattoirs from the two main pastoral regions of Ethiopia, namely Awash-Fentale pastoral area (Kereyu and Afar in the Middle Awash region) and Borena pastoral area (southern Ethiopia). The catchment areas possess large number of camels, in Fentale pastoral area (Middle Awash region) there are 68,331 camels and in Borena pastoral area of Oromia Regional State which border with Kenya possesses an estimated population of 97,131 camels [13]. After arriving at the abattoir, the camels were staying for 2–7 days undergoing physical examination. On average 6–8 camels were slaughtered per day depending on the request from customers. The main consumers of camel meat in Addis Ababa are the Somali immigrants residing in the city.

### Post mortem inspection and pathology scoring

Postmortem inspection was performed following the procedure as previously described [14]. Mandibular, retropharyngeal, bronchial, mediastinal, mesenteric and hepatic lymph nodes were examined and organs including lungs, liver, small intestine and kidneys were examined in detail during post-mortem in the abattoir under a bright-light source. The lobes of the left and right lungs were inspected and palpated externally. Then, each lobe was sectioned into about 2-cm-thick slices to facilitate the detection of lesions with sterile surgical blades. Similarly, lymph nodes were sliced into thin sections (about 2mm thick) and inspected for the presence of visible lesions. Whenever gross lesions suggestive of TB were detected in any of the tissue, the tissue was classified as having lesions.

Pathology scoring was conducted on tissues with abscesses and tubercle lesions to determine the severity of the lesions based on semi quantitative procedure developed previously [15], [16]. Briefly, lesions in the lobes of the lungs were scored separately as follows: 0 = no visible lesions; 1 = no gross lesions but lesions apparent on slicing of the lobe; 2 = fewer than five gross lesions; 3 = more than five gross lesions; 4 = gross coalescing lesions. The scores for the individual lobes were summed and generated lung score. Similarly, the severity of gross lesions in individual lymph nodes was scored as follows: 0 = no gross lesions; 1 = small lesion at one focus; 2 = small lesions at more than one focus; 3 = extensive necrosis. Individual lymph node scores were summed and generated the lymph node score. Total pathology score per animal was obtained from the sum of the two total scores.

### Mycobacterial isolation from tissue lesions

For mycobacteriological isolation tuberculous lesions from slaughtered camels were aseptically collected into sterile universal bottles with about 5 ml of 0.9% saline solution and also kept in icebox with solid packs to keep the cold chain. Then the samples were transported to Aklilu Lemma Institute of Pathobiology (ALIPB) and stored at +2 to +8°C until mycobacteriological culturing was carried out in TB laboratory.

The samples were further processed for isolation of mycobacteria in accordance with the Office International des Epizooties [17], [18]. The specimens were sectioned using sterile blades, minced with scissors and homogenized with a sterile mortar and pestle under a biological safety cabinet. The homogenates were decontaminated by adding an equal volume of 4% NaOH on the sample in order to remove contaminants. Thereafter, centrifuged at 3,000 rpm for 15 minutes to concentrate the mycobacteria. The supernatant was discarded, and the sediment was neutralized by 1% (0.1 N) HCl acid using phenol red as an indicator. Neutralization was achieved when the color of the solution changed from purple to yellow [17]. Next, 0.1 ml of suspension from each sample was spread onto a slant of Löwenstein Jensen (LJ) medium. Duplicate slants were used, one enriched with sodium pyruvate and the other enriched with glycerol. Cultures were incubated aerobically at 37°C for 8–12 weeks with weekly observation for growth of colonies. Positive cultures were confirmed with Ziehl Nelseen staining and preserved with freezing media while at the same time heat killed in water bath at 80°C for 45 minutes. The frozen and heat killed isolates were stored at (−20°C) for further mycobacteriology and molecular typing analysis.

### Mycobacterial genus typing

The multiplex PCR differentiate *M. tuberculosis* complex from *M. avium* complex, *M. intracellularae* and other mycobacterial species. Mycobacterial genus typing was conducted as described previously [19]. Heat killed AFB positive samples were used as source of DNA template.

DNA amplifications was done in thermocycler with 20 µl reaction volumes consisting: 5 µl of genomic DNA as a template, 8 µl HotstarTaqMasterMix (MgCL2, dNTP, Taq polymerase and PCR buffer) (Qiagen, United Kingdom) for each sample, 0.3 µl internal primer per sample, 0.3 µl forward and reverse primer per each sample and 5.2 µl per sample of Qiagen water. The primers used for amplification were MYCGEN-F, 5′AGA GTT TGA TCC TGG CTC AG 3′ (35ng/µl); MYCGEN-R, 5′TGC ACA CAG GCC ACA AGG GA 3′ (35ng/µl); MYCAV-R, 5′ ACC AGA AGA CAT GCG TCT TG 3′(35ng/µl); MYCINT-F, 5′CCT TTA GGC GCA TGA TGT CTT TA 3′(75ng/µl); TB1-F, 5′ GAA CAA TCC GGA GTT GAC AA 3′ (20ng/µl); TB-1-R, 5′ AGC ACG CTG TCA ATC ATG TA 3′ (20ng/µl). *M. tuberculosis* strains (H37Rv) and *M. avium* were used as positive control while Qiagen water was used as negative control. The reaction mixture was then heated in Programme Thermal Controller (Applied biosystem; PTC- 100™) cycle using the following amplification program: 95°C for 10 minutes for enzyme activation; 95°C for 1 minute for denaturation; 61°C for 0.5 minute for annealing; 72°C for 2 minutes for extension, involving 35 cycles all in all; and final extension at 72°C for 10 minutes.

The products were electrophoresed in 1% agarose gel in 10× TAE running buffer. Ethidium bromide at ratio of 1∶ 10, 100bp DNA ladder, and orange 6× loading dye were used in gel electrophoresis. All members of the genus *Mycobacterium* produce a band of 1030bp, *M. avium* or subspecies such as *M. avium* subspecies *paratuberculosis* produces a band of 180bp, *M. intracellularae* a band of 850bp while members of *M. tuberculosis* complex produce a band with 372bp.

### RD4 deletions typing

PCR analysis on the basis of RD regions has been found to be an important differentiating tool between members of the *M. tuberculosis* complex. RD4 is 12.7 kb genetic segment that is deleted from *M. bovis* BCG strain, but present in *M. microti*, *M. africanum*, and *M. tuberculosis* [20].

The RD4 deletion typing was carried out on isolates that showed band for *M. tuberculosis* complex by multiplex PCR. For this deletion typing, the procedure described by Cadamus and coauthors was followed [21]. Each sample was tested in a separate PCR tube. Primers directed against the RD4 were used to generate a deletion profile that would allow species identification of the isolate. Primers that were used include RD4intF ACA CGC TGG CGA AGT ATA GC, RD4flankF CTC GTC GAA GGC CAC TAA AG and RD4flankR AAG GCG AAC AGA TTC AGC AT to check for the presence of RD4 locus. The HotStarTaq Master Mix system from Qiagen was used for PCR, with primers described previously. The reaction mixture was 10 µl of HotStarTaq Master Mix, 0.3 µl×3 of each primer (flank R, F and int), 2 µl DNA template and 7 µl distilled water to a final volume of 20 µl. *M. tuberculosis* H37Rv and *M. bovis* 2122/97 were used as positive control while Qiagen water was used as negative control. The mixture was heated in Programme Thermal Controller (Applied biosystem; PTC- 100™) using an initial hot start of 95°C for 15 minutes, followed by 35 cycles of 95°C for 1 minute, 55°C for 1 minute, and 72°C for 1 minute; a final extension step of 72°C for 10 minutes to complete the cycle. PCR products were electrophoresed in 1% agarose gel in 1× TAE running buffer, Ethidium bromide at ratio of 1∶ 10, 100bp DNA ladder and orange 6× loading dye were used in electrophoresis. The gel was visualized in Multi–image™ light cabinet using Alpha innotech version 1.2.0.1(Alpha Innotech Corporation). The presence of RD4 (*M. tuberculosis*, *M. africanum*) gives a product size of 335bp (RD4int+RD4FlankR) and its absence (*M. bovis*) gives a product size of 446bp (RD4FlankR+RD4FlankF).

### Spoligotyping

Spoligotyping was performed as previously described by Kamerbeek and coauthors [22] and according to the spoligotype kit supplier's instructions (Ocimum Biosolutions Company, Iisselstein, The Netherlands). The direct repeat (DR) region was amplified by PCR using oligonucleotide primers derived from the DR sequence. A total volume of 25 µl the following reaction mixture was used for the PCR: 12.5 µl of HotStarTaq Master Mix (Qiagen: this solution provides a final concentration of 1.5 mM MgCl2 and 200µM of each deoxnucleotides triphosphates), 2 µl of each primer (20 pmol each), 5 µl suspension of heat-killed cells (approximately 10 to 50ng), and 3.5 µl distilled water. The mixture was heated for 15 minutes at 96°C and then subjected to 30 cycles of 1 minute at 96°C, 1 minute at 55°C, and 30 seconds at 72°C. The amplified product was hybridized to a set of 43 immobilized oligonucleotides, each corresponding to one of the unique spacer DNA sequences within the DR locus. After hybridization, the membrane was washed twice for 10 minutes in 2× SSPE (1× SSPE is 0.18 M NaCl, 10 mM NaH2PO4, and 1 mM EDTA[pH 7.7])-0.5% sodium dodecyl sulfate at 60°C and then incubated in 1∶4000 diluted streptavidin-peroxidase (Boehringer) for 45 to 60 minutes at 42°C. The membrane was washed twice for 10 minutes in 2× SSPE-0.5% sodium dodecyl sulfate at 42°C and rinsed with 2× SSPE for 5 minutes at room temperature. Hybridizing DNA was detected by the enhanced chemiluminescence method (Amersham) and by exposure to X-ray film (Hyperfilm ECL, Amersham) as specified by the manufacturer.

### Data management and analysis

Data were classified, filtered and coded using MS Excel 5, and was transferred to STATA version 8 for statistical analysis. Mean and standard error of the mean were used to summarize pathology scores. Similarly, proportions were used to summarize categorical exposure and outcome measures. Friedman test was used to compare pathology score of tropism of TB lesions among lymph nodes as well as among lung lobes. Bivariate and multivariable logistic regression analyses were used to assess the strength of associations of selected factors and prevalence of camel TB. Effects were reported as statistically significant if p-value was less than 5%. Odds ratio and 95% confidence intervals were used to measure the strength of associations.

## Results

### Prevalence of camel tuberculosis

On the basis of gross pathology, the prevalence of camel TB was 10% (91/906). Culture positivity was confirmed in 34% (31/91) of the camels with suspicious TB lesions. The result of the association of the different risk factors to the pathology showed that having a good body condition has a protective effect against being positive for TB (Table 1).

**Table 1:** Logistic regression analysis of tuberculous lesions with various host-related risk factors.

Characteristics	No. examined	No. of positive (%)	Crude Odds ratio (95% CI)	Adjusted Odds ratio (95% CI)
Sex				
Female	371	46 (12.4)	1	1
Male	535	45(8.4)	0.62 (0.40–0.95)	0.64 (0.36–1.2)
Age				
<4	99	12 (12.1)	1	1
4–6	184	14 (7.6)	0.60 (0.26–1.35)	0.25 (0.26–1.35)
7–9	141	10 (7.1)	0.55 (0.23–1.34)	0.54 (0.22–1.32)
10–15	197	19 (9.6)	0.77 (0.36–1.67)	0.64 (0.28–1.46)
16+	285	36 (12.6)	1.05 (0.52–2.11)	0.74 (0.32–1.68)
BCS				
Poor	389	44 (11.3)	1	1
Medium	330	36 (10.9)	0.96 (0.60–1.53)	0.92 (0.57–1.47)
Good	187	11 (5.9)	0.46 (0.25–.0.97)	0.42 (0.20–0.86)
Origin				
Kereyu	609	56 (9.2)	1	1
Borena	297	35 (11.8)	1.32 (0.84–2.06)	1.24 (0.70–2.2)

CI = Confidence Interval; BCS = Body Condition Scoring; odds ratio corresponding to different categories of a given variable are adjusted for the remaining three variables.

### Pathology scoring

**Figure 1:**
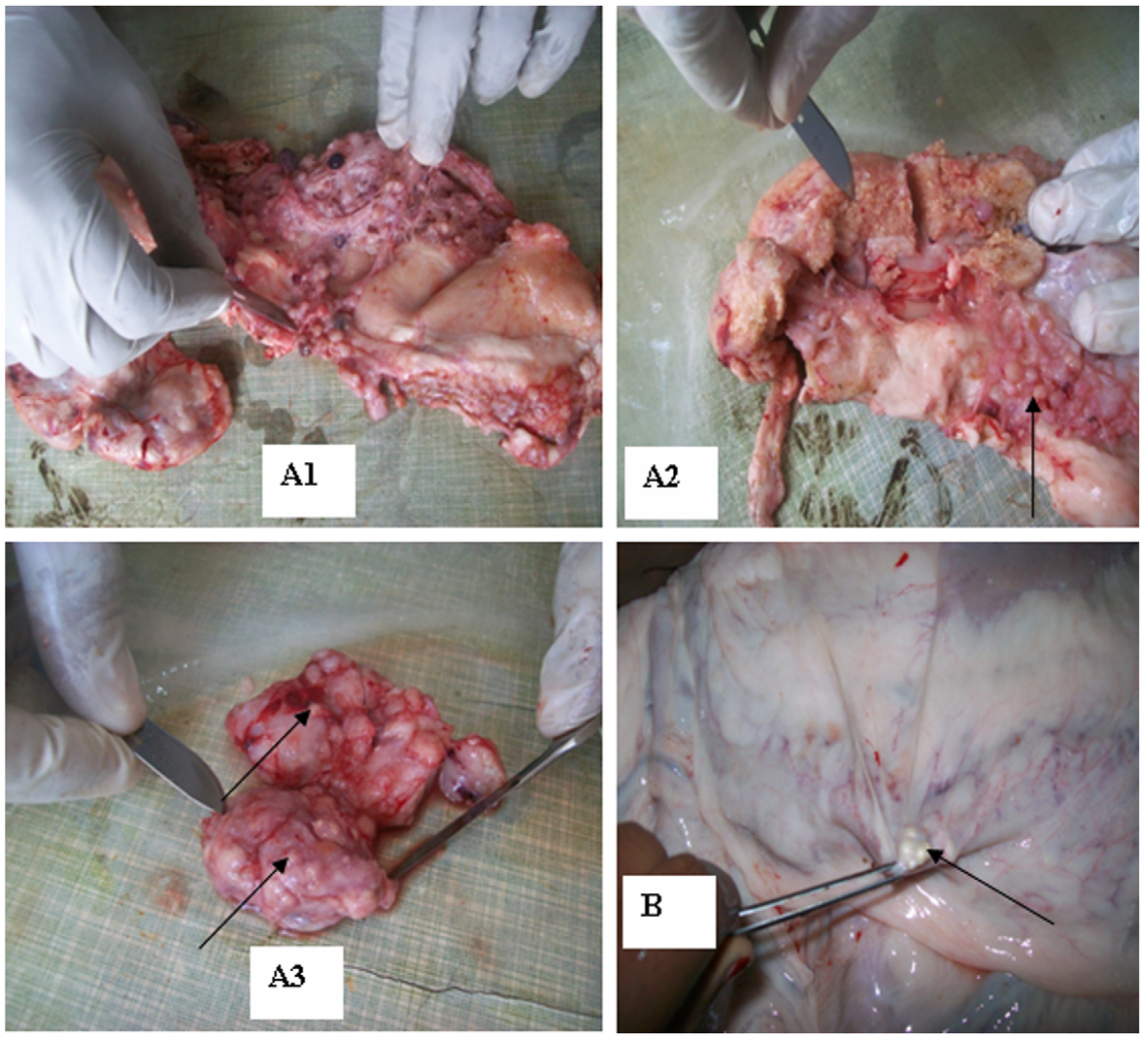
Tuberculous lesions from camels on different organs. (A1) Disseminated and distinct tuberculous lesions in mediastinal parts of the lung. (A2) Tuberculous lesion in mediastinal lymph node and nodules on other parts as indicated by arrows. (A3) Tuberculous lesions in hepatic lymph node. The arrows show that pea-sized lesions throughout the lymph node. (B) Tuberculous lesion in mesenteric lymph nodes as indicated by arrow.

The distribution of lesions and the severity of the disease were established in the 91 camels with suspicious lesions. The tropism of TB lesions to specific lymph nodes and lung lobes was statistically significant among the lymph nodes (χ^2^ = 22.697; P = 0.002) and lung lobes (χ^2^ = 17.901; P = 0.006) (Table 2). Lung lesions were detected in 43 camels while 78 camels had at least one lesion in their lymph nodes. The lesions appeared more frequent in the apical and cardiac lobes of both lungs than in the diaphragmatic lobes (Table 2). Similarly, the severity was greater in both right apical and cardiac lobes. Regarding lymph nodes, mesenteric lymph nodes were found the most frequently and severely affected of all the lymph nodes (34%) (Table 2, Figure 1).

**Table 2:** Distribution and tropism of tuberculous lesions in the lymph nodes and lung lobes in 91 postmortem positive camels with lesions in at least one tissue or organ.

	No (%) of camels with TB lesions			
Tissue	Total	Positive organs or tissue	χ^2^	P-value
**Lymph nodes**			22.697‡	*0.002
Parotid	91	13 (14.3%)		
Mandibular	91	15 (16.5%)		
Retropharyngeal	91	17 (18.7%)		
Mediastinal	91	30 (33%)		
Left bronchial	91	17 (18.7%)		
Right bronchial	91	21 (23.1%)		
Mesenteric	91	31 (34.1%)		
Hepatic	91	3 (3.3%)		
**Lung lobes**			17.901‡	*0.006
Left apical	91	30 (33%)		
Left cardiac	91	27 (29.7%)		
Left diaphragmatic	91	22 (24.2%)		
Right apical	91	25 (27.5%)		
Right cardiac	91	27 (29.7%)		
Right diaphragmatic	91	19 (20.9%)		
Right accessory	91	18 (19.8%)		

*Statistically significant.

‡ Chi-square was calculated from the median of pathology score of among tissues examined.

The mean severity of pathology of camel TB is summarized in Table 3. The mesenteric lymph node constituting the most severely affected lymph node (0.64±0.11; 0.55±0.15) followed by mediastinal lymph node (0.27±0.08).

**Table 3:** Mean pathology and standard error of the mean scoring of the lungs and lymph nodes of camels.

Lung lobes	Mean ± SEM	Lymph nodes	Mean ± SEM
Left apical lobe	0.64±0.11	Parotid	0.27±0.08
Left cardiac lobe	0.63±0.12	Mandibular	0.34±0.09
Left diaphragmatic lobe	0.56±0.11	Retropharyngeal	0.30±0.08
Right apical lobe	0.69±0.13	Mediastinal	0.55±0.15
Right cardiac lobe	0.72±0.13	Left bronchial	0.31±0.08
Right diaphragmatic lobe	0.47±0.11	Right bronchial	0.44±0.09
Right accessory lobe	0.41±0.10	Mesenteric	0.64±0.11

SEM = Standard Error of the Mean.

### Mycobacteriology

**Figure 2:**
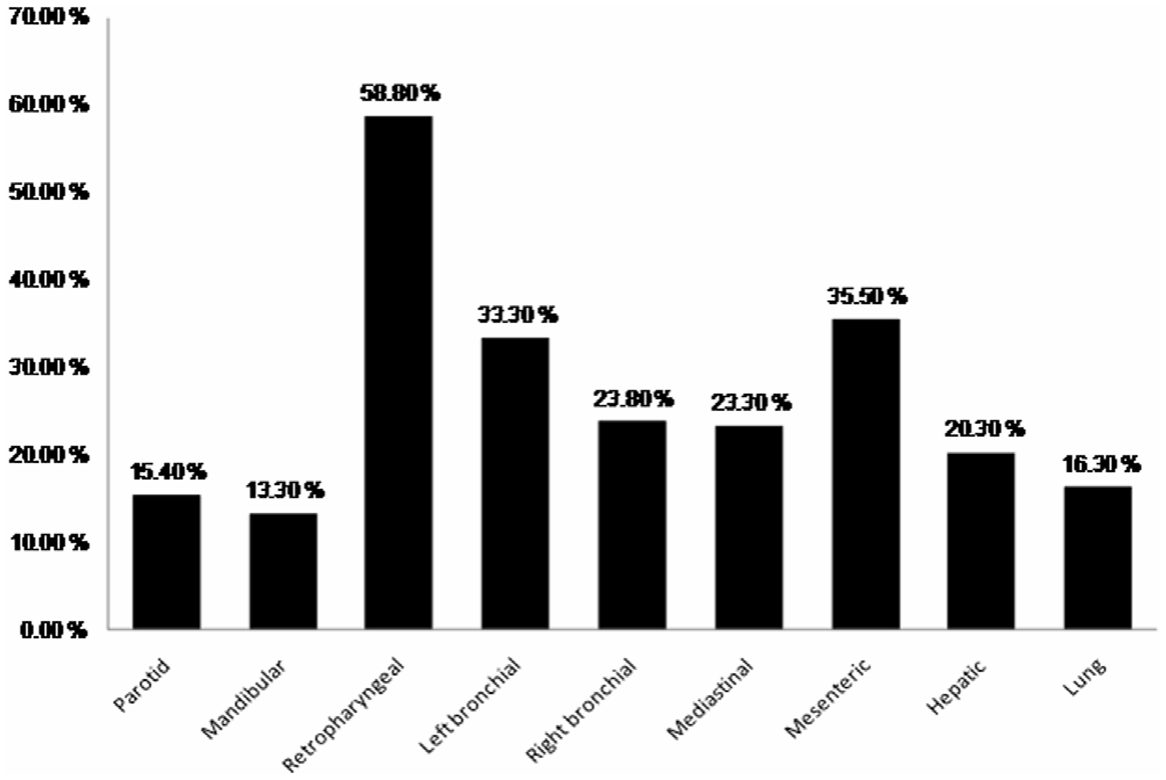
Proportion of mycobacterial culture positivity of the lymph nodes and lungs of TB suspected camels.

Growth of mycobacteria was observed in 34% (31/91) of camels with suspicious TB lesion (see Figure 2). Culture positivity was highest (58.8%) in the retropharyngeal lymph node followed by the mesenteric lymph node (35.5%). In contrast, isolation from mandibular and parotid lymph nodes were less frequently mycobacterial culture positive with the positivity of 13.3% and 15.4%, respectively.

### Molecular characterization of the isolates

#### Multiplex PCR

**Figure 3:**
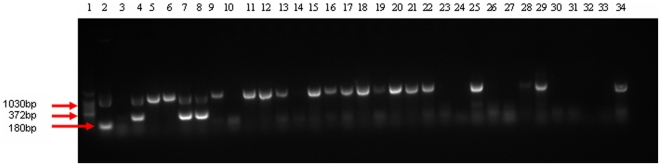
Gel electrophoresis separation of PCR products by multiplex PCR genus typing of mycobacteria isolated from naturally infected camels. Lane 1 = 100bp DNA Ladder; Lane 2 = *Mycobacterium avium* complex (positive control), Lane 3 = Qiagen H_2_O (negative control), Lane 4 = *Mycobacterium tuberculosis* complex (positive control), Lanes 5–34 were isolates from individual camels with tuberculous lesions. Lane 7 (sample 63), Lane 8 (sample 62) were positive for *Mycobacterium tuberculosis* complex and Lane 5, 6, 7, 8, 9, 11, 12–13, 15–22, 25, 28, 29, 34 were positive for genus Mycobacterium, Lane 10, 14, 23, 24, 26, 27, 30–33 were negative for genus Mycobacterium.

Further *Mycobacterium* genus typing was conducted on the 31 culture isolates from camels. Based on multiplex PCR using the primers of the *M. tuberculosis* complex and *M. avium* complex, 21 isolates gave signal to the genus *Mycobacterium*. Two of these isolates were confirmed to be members of the *M. tuberculosis* complex and none of the isolates were *M. avium* complex (Figure 3).

#### RD4 deletion typing

**Figure 4:**
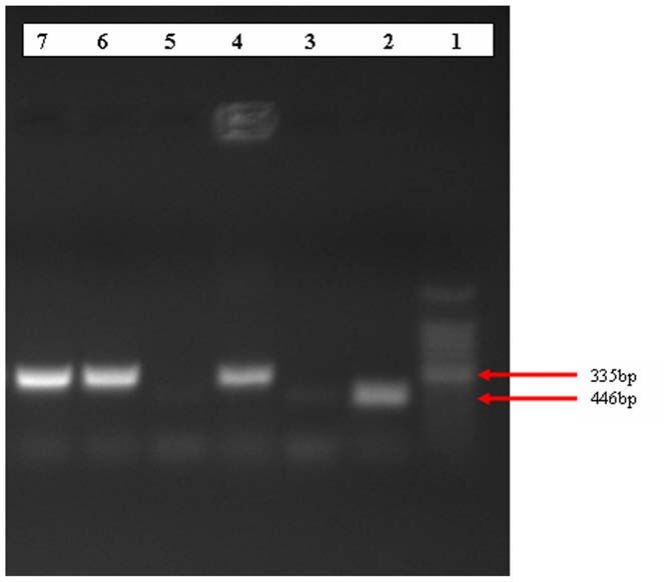
Gel electrophoresis separation of PCR products by RD4 deletion typing of mycobacteria isolated from naturally infected camels. Lane 1 = 100bp DNA ladder, Lane 2 = *M. tuberculosis* positive control, Lane 3 = Qiagen H_2_O (negative control), Lane 4 = *M. bovis* positive control, Lane 5–7 were isolates from camel, Lane 6 and 7 were positive for *M. bovis*.

The two isolates that showed signal to *M. tuberculosis* complex were subjected to RD4 deletion typing for further differentiation of species and they were confirmed to be *M. bovis* (Figure 4).

#### Spoligotyping

**Figure 5:**
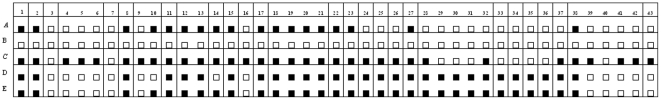
Schematic representation of the spoligotyping patterns of isolates of *Mycobacterium bovis* from camels with tuberculous lesions. A = *M.bovis* SB1176 (positive control); B = Qiagen H_2_O (negative control); C = *M. tuberculosis* (positive control); D = sample 63 (SB1953-New strain); E = sample 62 (SB0133). The black rectangles represent positive signals, and the white rectangles indicate negative signals.

The two isolates that showed signal with RD4 deletion PCR typing were further characterized using spoligotyping. One of these confirmed to be SB0133 and the other one was a new strain which was not reported previously in *M. bovis* database. The new strain was reported to the global database (http://www.Mbovis.org) and designated as SB1953 (Figure 5). The SB0133 isolate was isolated from camel with generalized and disseminated form of TB.

## Discussion

In general, there is scanty information on TB in camels. Nonetheless, there are few reports published on camel TB in Ethiopia as well as in other countries. The prevalence of camel TB recorded by the present study is similar to the report of previous study in the Afar Region of Ethiopia based on comparative intradermal tuberculin test in camels [23] but it is higher than the report from Dire Dewa Abattoir in camels from eastern Ethiopia [12]. Similarly, it is higher than the prevalence reported in Egypt [6].

The occurrences of TB lesions in camels were relatively higher in the younger and older camels than other age groups. Other researchers have also reported in cattle particularly that older animals are affected by TB [24]–[27] which could be due to the fact that older animals have weaker immune system. The higher frequency of lesion in younger camels could be due to the less developed immunity [28]. Young camels can also be easily infected with higher doses of mycobacteria via colostrums from infected camel in a similar way, as it occurs in cattle [29]. In connection with this, another report mentioned of vertical transmission of *M. bovis* from an infected dam to her calf through congenital infection in utero [30]. It was observed that lesion was more frequently observed in female camels as compared to male camels. This could be due to the fact that female camels were brought for slaughter at their older age after completion of the reproductive age [26], [31].

The distribution, frequency, and severity of lesions recorded in different tissues of camels were similar with the reports of similar studies in grazing cattle in Ethiopia [18], [32]. In these studies, the frequency and severity of the lesions were higher in the mesenteric lymph nodes than the thoracic lymph nodes, while in other studies under intensive cattle husbandry lesions were predominant in the respiratory tract and thoracic lymph nodes [14], [16].

Tuberculous lesions were subjected to bacteriological culture so as to identify and characterize the causative agents. However, culture positivity of suspicious tissues was 34%, which is lower than what have been reported previously from cattle [18], [32]. The lower culture positivity might be related to the non-optimal condition of the culture for NTM which assumed to be the major isolates causing pathology in camel. Regarding culture positivity of each organ, the highest culture positivity was recorded in the retropharyngeal lymph node followed by mesenteric lymph node, which could suggest that oral route could be the main route of infection. In contrast, other authors have reported that culture positivity was higher in lung tissue and thoracic lymph nodes than in the head and mesenteric lymph nodes [14], [32], [33].

Genus typing of the isolates revealed that out of 21 isolates which showed signals for the genus *Mycobacterium*, only two isolates were *M. bovis* as confirmed by RD4 deletion typing and spoligotyping, while the remaining 18 did not show signal to the *M. tuberculosis* complex, and hence assumed to be members of nontuberculous mycobacteria (NTM). In the present study, the NTM resulted sarcoid-like tuberculous nodules with granulomatous and caseous lesions in lymph nodes, lung and other organs of camel. Previous study reported also the isolation NTM including *M. kansasii* and *M. smegmatis* from tuberculous like lesions in camel causing a similar caseous nodules like those caused by *M. bovis* and *M. tuberculosis* [11]. In Ethiopia, NTM have been isolated from cattle with tuberculous lesions in different regions of the country [34], which indicates their wider geographic distribution and role as a cause of tuberculous lesions in livestock of the country. Therefore, further identification and characterization of these isolates are necessary.

Spoligotyping of the two *M. bovis* isolates revealed a distinct spoligopattern. Referring to the global http://www.Mbovis.org database of the spoligopatterns indicated that one of the strains which caused a generalized disseminated TB in camel was SB0133, whereas the other strain was new strain not reported in the database previously. The new strain now has been reported to the database and designated as SB1953. In Ethiopia, a number of studies reported new strains with specific spoligotype pattern in cattle [32], [34]–[36]. The identification of this new *M. bovis* strain from camel TB case of pastoral area of Ethiopia indicates the need for further research in identifying the circulating strains of *M. bovis* in various hosts and their distribution in geographical area of the country. On the other hand, the isolation of SB0133 *M. bovis* strain in present study from camel of pastoral area of Ethiopia inline with the isolation of this strain from cattle of southern Ethiopia [34], [36] and pastoral area of Uganda [37] indicates the predominant localization of the strain to pastoral regions of Eastern Africa and possible interspecies transmission of the strain among livestock of pastoral area. In addition, the development of TB lesions to generalized disseminated form of TB in camel affected by SB0133 strain might imply its high pathogenicity in camels.

In conclusion, the present study has shown that the majority of camel TB lesions were caused by NTM; hence, further identification and characterization of these species would be useful towards the efforts made to control TB in camels. The isolation of *M. bovis* strain (SB0133) which is similar to cattle strain in pastoral area of East Africa, implies the existence of potential inter-species transmission of the strain among livestock of pastoral area, warrant further investigation to elucidate its epidemiological significance for public health and control of the disease in the region.
